# CD86^+^/CD206^+^, Diametrically Polarized Tumor-Associated Macrophages, Predict Hepatocellular Carcinoma Patient Prognosis

**DOI:** 10.3390/ijms17030320

**Published:** 2016-03-01

**Authors:** Pingping Dong, Lijie Ma, Longzi Liu, Guangxi Zhao, Si Zhang, Ling Dong, Ruyi Xue, She Chen

**Affiliations:** 1 Key Laboratory of Glycoconjugate Research Ministry of Public Health, Department of Biochemistry and Molecular Biology, Shanghai Medical College, Fudan University, Shanghai 200032, China; 13211210002@fudan.edu.cn (P.D.); 14211210033@fudan.edu.cn (L.M.); 15111210063@fudan.edu.cn (L.L.); 14111210029@fudan.edu.cn (G.Z.); zhangsi_1@aliyun.com (S.Z.); 2 Department of Gastroenterology and Hepatology, Zhongshan Hospital, Fudan University, Shanghai 200032, China; dong.ling@zs-hospital.sh.cn; 3 Shanghai Institute of Liver Diseases, Zhongshan Hospital, Fudan University, Shanghai 200032, China; 4 Department of Hepatic Surgery, Liver Cancer Institute, and Key Laboratory of Carcinogenesis and Cancer Invasion (Ministry of Education), Zhongshan Hospital, Fudan University, Shanghai 200032, China

**Keywords:** hepatocellular carcinoma, tumor-associated macrophages, CD86, CD206, AFP, prognosis

## Abstract

Tumor-associated macrophages (TAMs), the most abundant infiltrating immune cells in tumor microenvironment, have distinct functions in hepatocellular carcinoma (HCC) progression. CD68^+^ TAMs represent multiple polarized immune cells mainly containing CD86^+^ antitumoral M1 macrophages and CD206^+^ protumoral M2 macrophages. TAMs expression and density were assessed by immunohistochemical staining of CD68, CD86, and CD206 in tissue microarrays from 253 HCC patients. Clinicopathologic features and prognostic value of these markers were evaluated. We found that CD68^+^ TAMs were not associated with clinicopathologic characteristics and prognosis in HCC. Low presence of CD86^+^ TAMs and high presence of CD206^+^ TAMs were markedly correlated with aggressive tumor phenotypes, such as multiple tumor number and advanced tumor-node-metastasis (TNM) stage; and were associated with poor overall survival (OS) (*p* = 0.027 and *p* = 0.024, respectively) and increased time to recurrence (TTR) (*p* = 0.037 and *p* = 0.031, respectively). In addition, combined analysis of CD86 and CD206 provided a better indicator for OS (*p* = 0.011) and TTR (*p* = 0.024) in HCC than individual analysis of CD86 and CD206. Moreover, CD86^+^/CD206^+^ TAMs predictive model also had significant prognosis value in α-fetoprotein (AFP)-negative patients (OS: *p* = 0.002, TTR: *p* = 0.005). Thus, these results suggest that combined analysis of immune biomarkers CD86 and CD206 could be a promising HCC prognostic biomarker.

## 1. Introduction

With an increasing incidence rate, hepatocellular carcinoma (HCC) is ranked the second cause of cancer-related deaths around the world [1]. Currently, surgical resection is the preferred treatment for HCC. However, large cohorts of HCC patients suffer from postoperative recurrence, and have a poor response to systemic chemotherapeutic treatments, with a five-year survival rate of only 30%–40% [2]. Still worse, current clinicopathologic factors, such as α-fetoprotein (AFP), tumor-node-metastasis (TNM) stage, and Barcelona clinic liver cancer (BCLC) stage, cannot accurately predict the outcome of HCC patients. Novel prognostic markers need to be developed for more customized HCC treatment. HCC is a typical inflammation-related cancer. Chronic inflammation provides a favorable surrounding to facilitate HCC progression [3,4]. Accumulating evidence indicates that tumor microenvironment plays a vital role in tumor progression and metastasis [5]. HCC microenvironment could be rich resources for identifying novel powerful prognostic biomarkers.

Macrophage is a main cellular ingredient in human tumor microenvironment, and is commonly known as tumor-associated macrophages (TAMs) [6]. Different types of macrophage have distinct functions in tumor progression. M1 macrophages, which activate tumor-killing mechanisms, as well as amplify Th1 immunocytes responses, provide a resistant role in tumorigenesis. On the other hand, M2 macrophages, via suppressing tumor-specific immune responses, mainly act to enhance tumor growth and metastasis [7].

Accumulating evidence indicates that CD68 was expressed in all macrophages, and is labeled as a pan-macrophage biomarker [8]. However, CD68 cannot effectively distinguish between M1 and M2 subtype macrophages. Previous study implied that M1 macrophages expressed high level of CD86 and tumor necrosis factor α (TNF-α), while M2 macrophages expressed relatively high level of CD206, CD163 and IL-10 [9,10]. Interestingly, Tan *et al.* reported that in HCC, M1 macrophages expressed increased level of CD86 relative to TNF-α and IL-12, while M2 macrophages expressed increased level of CD206 relative to IL-10, and transforming growth factor β (TGF-β) [11]. Recent studies have demonstrated that TAMs were associated with HCC progression, and may act as a promising prognostic factor and therapeutic target [12,13].

In this study, we showed that CD68^+^ TAMs alone had no prognostic value in HCC patients, indicating that total macrophages had no impact on HCC prognosis. Low presence of CD86^+^ and high presence of CD206^+^ TAMs were clearly correlated with aggressive tumor phenotypes, such as multiple tumor number and advanced TNM stage; and were associated with a poor prognosis in survival and recurrence. Furthermore, combined analysis of CD86 and CD206 provided a better prognostic indicator for HCC patients than individual analysis of CD86 and CD206. Furthermore, CD86^+^/CD206^+^ TAMs predictive model also showed strong prognosis value in AFP-negative patients.

## 2. Results

### 2.1. Characterization of Tumor-Associated Macrophages in Hepatocellular Carcinoma (HCC) Patients

**Figure 1:**
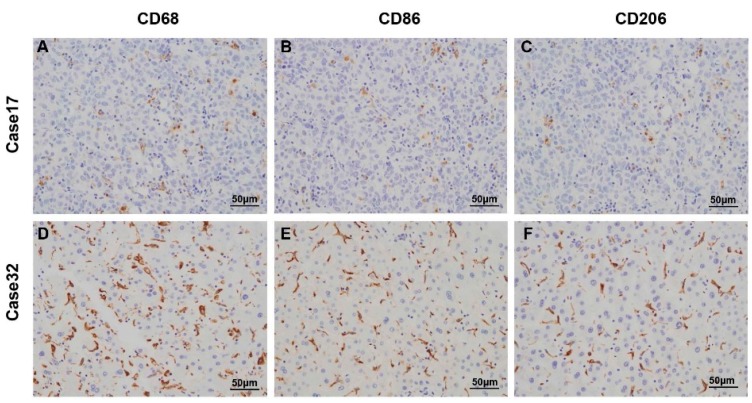

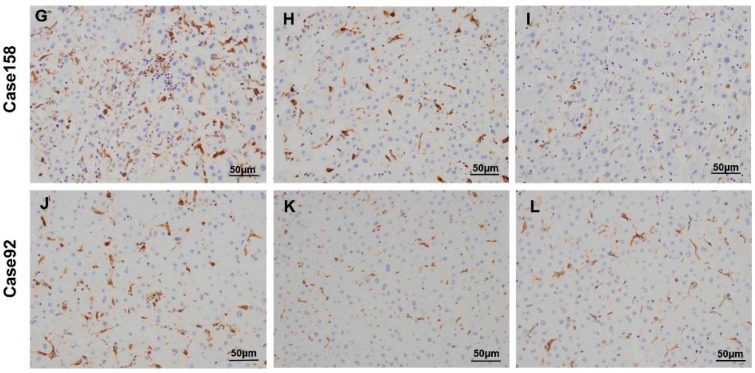
Representative immunostaining images of CD68 (**A**,**D**,**G**,**J**); CD86 (**B**,**E**,**H**,**K**); and CD206 (**C**,**F**,**I**,**L**) in HCC tissue microarray sections were shown. Case 17 (**A**–**C**) showed low staining presence of CD68^+^, CD86^+^ and CD206^+^ macrophages; Case 32 (**D**–**F**) showed high staining presence of CD68^+^, CD86^+^ and CD206^+^ macrophages; Case 158 (**G**–**I**) showed high staining presence of CD68^+^ and CD86^+^ macrophages, but low staining presence of CD206^+^ macrophages; Case 92 (**J**–**L**) showed high staining presence of CD68^+^ and CD206^+^ macrophages, but low staining presence of CD86^+^ macrophages.

**Figure 2:**
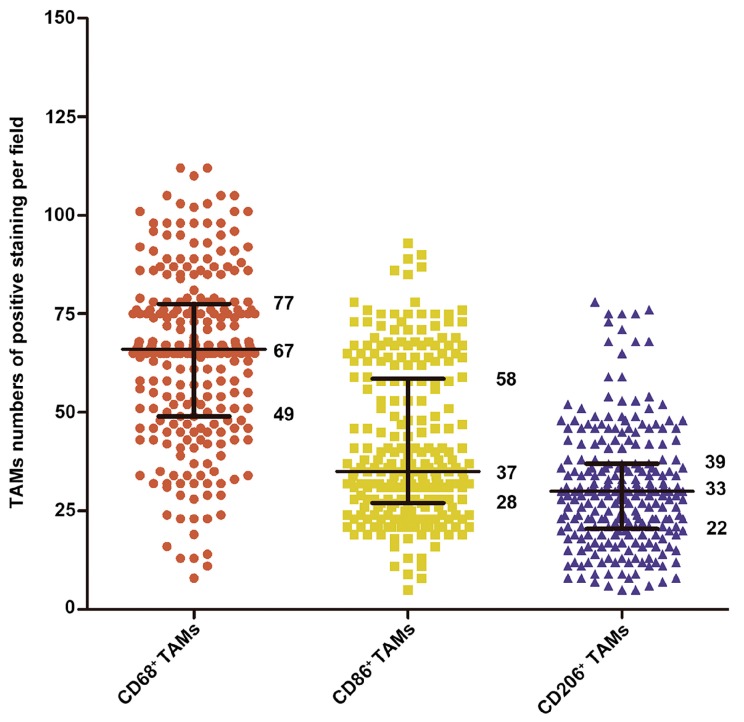
The number distribution of CD68^+^, CD86^+^ and CD206^+^ macrophages in the cohort (*n* = 253). Lines indicated 25th, 50th, and 75th percentiles. TAMs: Tumor-associated macrophages.

Immunohistochemistry was performed to assess the expression and presence of macrophages in tumor tissues from 253 HCC patients who had undergone curative resection. CD68, CD86, and CD206 positive staining were mainly located in the cytoplasm of macrophages (Figure 1). In tumor tissues, the number of CD68 positive cells (median, 67 cells/field) was higher than CD86 positive (median, 37 cells/field, *p* < 0.001) and CD206 positive cells (median, 33 cells/field, *p* < 0.001, Figure 2 and Table S1).

### 2.2. Association between Macrophage Markers Presence (CD68, CD86 and CD206) and Clinicopathologic Characteristics in HCC Patients

We next investigated the association between macrophage markers (CD68, CD86 and CD206) and patients’ clinicopathologic characteristics. The 253 patients were divided into two groups (low and high) based on the median value of CD68, CD86, and CD206 staining cells, respectively. As summarized in Table 1, CD68 positive staining count in tumor had no relationship with any clinicopathologic features. However, lower infiltration of CD86^+^ TAMs was associated with aggressive tumor phenotypes, such as multiple tumor number (*p* = 0.006), high-grade TNM stage (*p* = 0.001) and elevated alaninetransaminase (ALT) (*p* = 0.020). Interestingly, higher infiltration of CD206^+^ TAMs was also positively correlated with multiple tumor number (*p* = 0.038), presence of vascular invasion (*p* = 0.011), appearance of tumor capsulation (*p* = 0.004), and advanced TNM stage (*p* = 0.005).

### 2.3. Analysis of Macrophages Immune Marker (CD68, CD86 and CD206) Prognostic Value in HCC Patients

**Figure 3:**
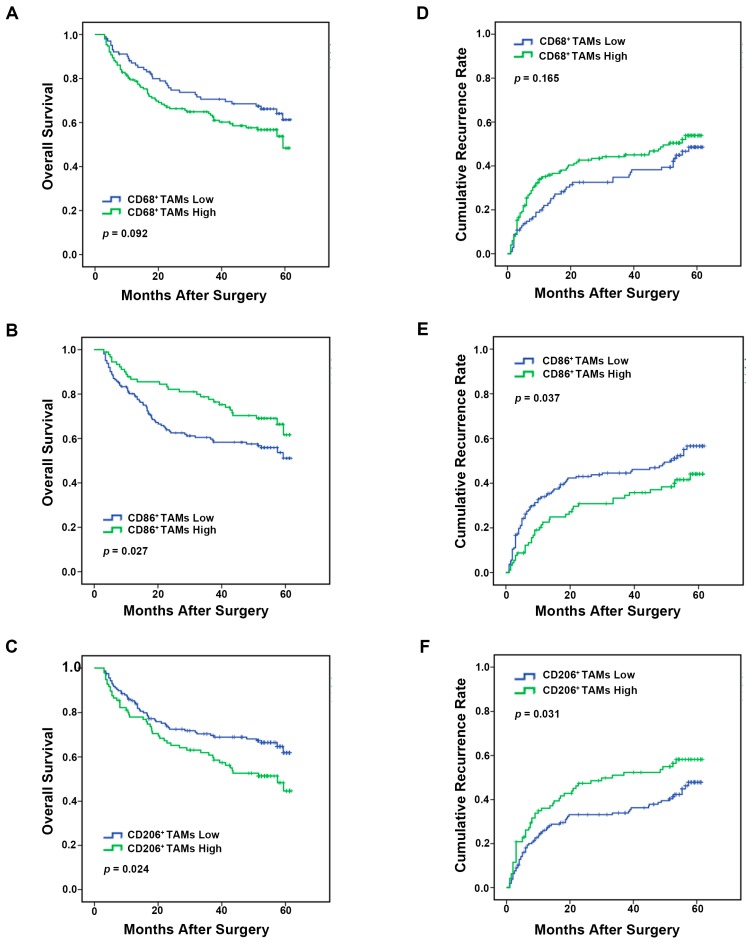
Kaplan–Meier curves for overall survival (**A**–**C**) and time to recurrence (**D**–**F**) of hepatocellular carcinoma (HCC) patients according to the staining presence of CD68^+^, CD86^+^ and CD206^+^ macrophages in the cohort (*n* = 253).

We further investigated the clinic prognosis value of TAMs markers in this cohort of 253 patients (Figure 2). CD68^+^ TAMs had no prognostic value in HCC patients (Figure 3A,D). Patients with low CD86^+^ TAMs staining cells had a significantly shorter median overall survival (OS) and time to recurrence (TTR) (OS, 41.3 months; TTR, 36.3 months) than those with high staining cells (OS, 49.1 months, *p* = 0.027; TTR, 43.2 months, *p* = 0.037) (Figure 3B,E). Conversely, low presence of CD206^+^ TAM group had a markedly longer median OS and TTR (OS, 46.2 months; TTR, 41.7 months) when compared with the high presence group (OS, 40.1 months, *p* = 0.024; TTR, 34.0 months, *p* = 0.031) (Figure 3C,F).

As summarized in Table 2, univariate analyses suggested that low CD86^+^ TAMs and high CD206^+^ TAMs were significantly associated with decreased survival and high risk of recurrence in HCC patients post curative resection. Furthermore, multivariate Cox’s regression analysis, after backward stepwise variable selections, suggested that apart from tumor size, tumor differentiation, and vascular invasion, infiltration of CD86^+^ and CD206^+^ TAMs remained as the independent prognostic factors in HCC patients for both OS (HR = 2.178, *p* = 0.040 and HR = 1.584, *p* = 0.027) and TTR (HR = 1.810, *p* = 0.006 and HR = 1.872, *p* = 0.030). Collectively, these data implied that CD86 and CD206 were valuable prognostic biomarkers in HCC patients.

### 2.4. Integrated Analysis of Immune Markers CD86 and CD206 Provides More Powerful Prognostic Value in HCC Patients

**Figure 4:**
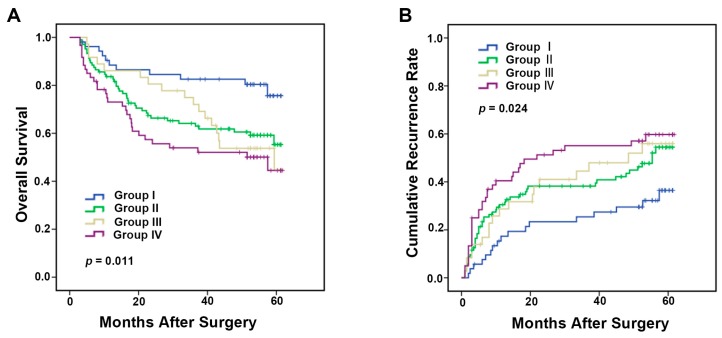
Kaplan–Meier curves for overall survival (**A**) and time to recurrence (**B**) according to the comprehensive analysis of the staining presence of CD86^+^ and CD206^+^ macrophages in the above cohort (*n* = 253). Group I, high staining presence of CD86^+^ but low CD206^+^ macrophages; Group II, both low staining presence; Group III, both high staining presence; and Group IV, low staining presence of CD86^+^ but high CD206^+^ macrophages.

As important tumor microenvironment components, antitumoral M1 and protumoral M2 immunophenotype macrophages both influence the tumor development and progression. Thus, we hypothesized that combined analysis of CD86 and CD206 may better predict the prognosis of HCC patients by evaluating both M1 and M2 immunophenotype macrophages. Based on CD86^+^ and CD206^+^ TAMs presence, patients were classified into four groups: Group I, CD86^high^ and CD206^low^; Group II, CD86^low^ and CD206^low^; Group III, CD86^high^ and CD206^high^; and Group IV, CD86^low^ and CD206^high^. The median OS for Groups I, II, III, and IV were 52.5, 43.0, 45.2 and 37.8 months, respectively (Figure 4A). The median TTR for Groups I, II, III, and IV were 47.4 months, 38.7, 37.4 and 31.9 months, respectively (Figure 4B). Significant differences in OS and TTR were found among the four groups (OS: *p* = 0.011, TTR: *p* = 0.024, Figure 4A,B). Collectively, these data evidently suggested that combined analysis of CD86 and CD206 served as a better indicator of survival and recurrence in HCC patients than analyzing individual factors.

### 2.5. TAMs Predictive Model for α-Fetoprotein (AFP) Negative HCC Patients

**Figure 5:**
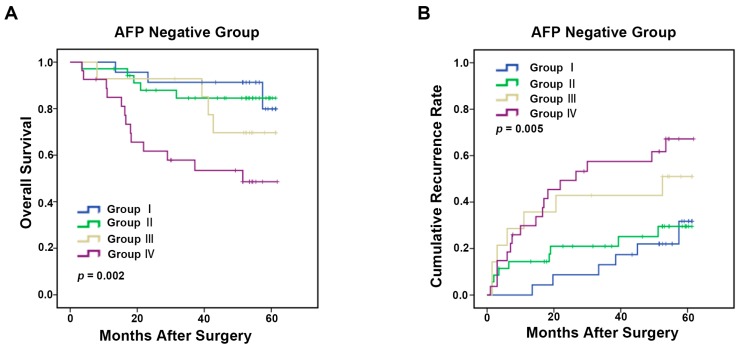
Kaplan–Meier curves for overall survival (**A**) and time to recurrence (**B**) of HCC patients with negative α-fetoprotein (AFP) (AFP ≤ 20 ng/mL) based on the CD86^+^/CD206^+^ TAMs predictive model in the cohort (*n* = 99).

Several clinical studies demonstrated that preoperative serum AFP level was a promising predictor for HCC prognosis [14,15,16]. Low AFP level is generally associated with favorable prognosis. Nevertheless, some patients with negative AFP developed rapidly. Still worse, there is no reliable biomarker to differentiate the prognosis of AFP^−^ HCC patients. To test the prognostic value of TAMs marker in AFP^−^ patients (cut-off point 20 ng/mL) [17,18], 99 patients were selected from as the abovementioned cohort. In this AFP^−^ cohort, patients with CD86^low^ and CD206^high^ status had the worse OS and TTR (OS: 40.5 months, *p* =0.002, TTR: 32.6 months, *p* = 0.005, Figure 5A,B) compared with three other groups (Group I, CD86^high^ but CD206^low^, OS: 57.1 months, TTR: 54.1 months; Group II, CD86^low^ and CD206^low^ , OS: 54.8 months, TTR: 48.8 months; Group III, CD86^high^ but CD206^high^ , OS: 52.8 months, TTR: 37.4 months).

## 3. Discussion

Tumor milieu consists of many cell types. Cross-talks between tumor cells and the ambient microenvironment plays pivotal role in tumor progression and metastasis. In particular, TAM is a major cellular component that infiltrates most tumors, establishing a bridge between tumor cells and immune microenvironment [19]. Accumulating evidence indicated that the subtypes of macrophages, M1 and M2 phenotypes, performed exactly opposite roles in tumor progression and metastasis [10,20,21]. M1 acted as a proinflammatory factor against microorganisms and tumor cells [22], while M2 played an immunosuppressive role to promote tissue repair and tumor progression [23]. In this study, CD68 was used as pan macrophages immune marker; while CD86 and CD206 as marker for M1 and M2, respectively. Other molecules like CD11c and CD163 are also, respectively, expressed in M1 and M2 macrophages, but are at lower level compared with the former two in HCC. This makes CD86 and CD206 promising candidates as prognostic biomarkers compared with other macrophage biomarkers.

This study indicated that the presence of CD68^+^ TAMs had no impact on HCC prognosis. This may attribute to functional counterbalance regulated by M1 and M2 macrophages. Previous studies in HCC, colon cancer and gastric cancer implied that the abundance of CD68^+^ TAMs infiltrated in tumor tissue was not associated with patient prognosis after curative cancer tissue resection [24,25,26]. However, other research reported that CD68^+^ TAMs in tumor stroma was an independent prognostic factor for poor OS and TTR in breast cancer, cholangiocarcinoma and Hodgkin lymphoma [27,28,29]. The relationship between CD68^+^ TAMs and tumor prognosis is not clear-cut. Different CD68^+^ TAMs polarization status may indicate different prognosis.

In contrast to CD68^+^ TAMs infiltration, the low presence of CD86^+^ TAMs and high presence of CD206^+^ TAMs markedly correlated with poor HCC prognosis. Although plentiful studies described CD86 and CD206 as a cell surface immune marker of M1 and M2 macrophage respectively, only a few reports emphasized the clinic significance of CD86^+^ and CD206^+^ TAMs in tumors. In colorectal cancer, the infiltration of CD86^+^ TAMs indicated a favorable prognosis [30]. In multiple myeloma patients, CD86^+^ TAMs did not show correlation with tumor progression [31]. In prostate adenocarcinoma, the high presence of CD206^+^ TAMs infiltration was associated with poor prognosis [32]. Similarly, Xu *et al.* reported that CD206^+^ TAMs was a promising indicator for poor survival in renal cell carcinoma patients [33]. In gastric cancer, infiltration of polarized CD206^+^ TAMs in tumor indicated poor survival after surgical resection [26]. These results, as well as findings from this study, indicated that polarized M1 and M2 play a vital role in tumor prognosis.

Many reports highlighted the prognostic values of individual M1 or M2 in cancers, but did not perform a combined analysis of M1 and M2. It should be noted that M1 and M2 macrophage polarization are the two ends of macrophages. Most macrophages take on a mixed M1/M2 phenotype [34,35]. Combined analysis of M1/M2 phenotype TAMs seems to be more appropriate in cancer patients. In the present study, patients from CD86^low^/CD206^low^ and CD86^high^/CD206^high^ groups had the intermediate OS and TTR, possibly attributed to functional counterbalance regulated by CD86 and CD206. CD86^high^/CD206^low^ group implied an immune profile of M1 macrophage polarization and served as a favorable prognostic factor in OS and TTR. On the other hand, CD86^low^/CD206^high^ group showed an immune profile of M2 macrophage polarization, and was associated with a poor HCC prognosis. Our results further emphasized the opposite functions of M1 and M2 macrophages in HCC prognosis.

Previous reports revealed that about 40% early-stage HCC patients and 15%–20% late-stage HCC patients were AFP-negative [36]. Generally, HCC patients with negative AFP were associated with favorable prognosis. However, some AFP^−^ patients progressed rapidly, with poor prognosis. Thus far, there is no satisfactory prognostic factor for AFP^−^ patients. It is urgent to develop a novel prognostic factor for patients with negative AFP. In our study, when applied to preoperative serum AFP-negative patients, CD86^low^ but CD206^high^ TAMs status could effectively differentiate patients with poor prognosis. Thus, to some extent, this predictive model could be a powerful tool for making rational treatment decision in AFP-negative patients.

In summary, this study indicated that CD86^high^/CD206^low^ group, implying M1 immunophenotype macrophages, and CD86^low^/CD206^high^ group, implying M2 immunophenotype macrophages, provides favorable and poor prognosis of HCC, respectively. Combined analysis of immune biomarkers, CD86 and CD206, may serve as a better prognostic indicator than individual analysis for HCC survival and recurrence, especially in AFP negative patients.

## 4. Experimental Section

### 4.1. Patients

Human HCC tissues were obtained from surgical specimens of liver cancer patients in Fudan University affiliated Zhongshan Hospital (Shanghai, China). We retrospectively collected 253 patients undergoing liver cancer surgical resection between October 2009 and February 2011. Collection of clinical data and post-operation follow-up were consistent with harmonized standard [37]. Overall survival (OS) was defined as the time from the date of surgery to death. Time to recurrence (TTR) was defined as the time from the date of operation to recurrence. However, if recurrence was not observed or patients were still alive at the last follow-up (April 2015), data were censored. This study was approved by ethics committee of Fudan University affiliated Zhongshan Hospital (permission date, 26 February 2015; permission code, 20150102), and informed consent was obtained from each patient.

### 4.2. Immunohistochemistry

Paraffin-embedded tissue microarray (TMA) sections were deparaffinized in xylene and rehydrated in a reducing ethanol concentration series diluted with distilled water. Antigen retrieval was conducted in a preheating antigen retrieval buffer (citrate buffer, pH = 6.0) for 20 min. The TMA sections were stained with a rabbit monoclonal anti-CD68 (1:100, Abcam, Cambridge, MA, USA), a rabbit monoclonal anti-CD86 (1:100, Abcam, Cambridge, MA, USA) and a rabbit monoclonal anti-CD206 (1:100, Abcam, Cambridge, MA, USA) overnight at 4 °C. After being washed with phosphate-buffered saline (PBS) containing 0.1% Tween 20 (PBST), the sections were incubated with secondary antibodies for 1 h at room temperature. The slides were colorated with DAB (3,3′-diaminobenzidine tetrahydrochloride), and redyed with haematoxylin. The TMA sections were observed under the CKX31 microscope (Olympus, Osaka, Japan). Positive staining cells were counted every field under high magnification (200×). Positive staining cells were defined as those stained with brown regardless of the color depth. For each patient, the mean number of three random fields was used for data analysis. The positive staining cells were counted by Image Pro Plus6.0 analysis software (Media Cybernetics, Bethesda, MD, USA), and the detailed procedure is described elsewhere [38]. Based on the median value of CD68, CD86, and CD206 positive staining cells, patients were divided into high and low group [39]. The assessment ware carried out by two independent researchers, blinded of patient outcome. The intra-observer reproducibility was tested by obtaining the widely used statistical κ-scores [40].

### 4.3. Statistical Analysis

Analysis of the association between macrophage markers (CD68, CD86, and CD206) expression and clinicopathological characteristics was carried out using Pearson’s Chi-square test. Kaplan–Meier analysis (log-rank test) was utilized for OS and TTR curves. Variables related to OS and TTR were analyzed using univariable Cox’s regression analysis. Significant factors were further analyzed using a multivariable Cox’s regression analysis model. SPSS software (20.0; SPSS, Inc., Chicago, IL, USA) and Graph Pad Prism 5.0 software (Graph Pad-Prism Software, San Diego, CA, USA) were used for data analyses. *p* value <0.05 was considered significant.

## Figures and Tables

**Table 1:** Correlation between immunohistochemical variables and clinicopathologic features of HCC patients in the cohort (*n* = 253).

Characteristics	CD68^+^ TAMs			CD86^+^ TAMs			CD206^+^ TAMs		
	Low	High	*p* ^#^	Low	High	*p* ^#^	Low	High	*p* ^#^
Age (years)									
≤51	50	81	0.522	86	45	0.602	80	51	0.796
>51	52	70	-	76	46	-	77	45	-
Gender									
Female	17	16	0.184	142	78	0.699	21	12	1.000
Male	85	135	-	20	13	-	136	84	-
HBsAg									
Negative	4	8	0.767	8	4	1.000	8	4	1.000
Positive	98	143	-	154	87	-	149	92	-
HCVAb									
Negative	101	146	0.406	158	89	1.000	153	94	1.000
Positive	1	5	-	4	2	-	4	2	-
AFP (ng/mL)									
≤20	44	55	0.296	61	38	0.592	58	41	0.426
>20	58	96	-	101	53	-	99	55	-
ALT (U/L)									
≤75	88	137	0.309	150	75	0.020	141	84	0.680
>75	14	14	-	12	16	-	16	12	-
γ-GT (U/L)									
≤54	47	64	0.606	67	44	0.294	72	39	0.436
>54	55	87	-	95	47	-	85	57	-
Liver cirrhosis									
NO	15	18	0.570	23	10	0.561	22	11	0.701
YES	87	133	-	139	81	-	135	85	-
Tumor size (cm)									
≤5	59	81	0.522	86	54	0.359	90	50	0.436
>5	43	70	-	76	37	-	67	46	-
Tumor number									
Single	82	118	0.753	137	63	0.006	131	69	0.038
Multiple	20	33	-	25	28	-	26	27	-
Vascular invasion									
No	65	95	1.000	106	54	0.345	109	51	0.011
Yes	37	56	-	56	37	-	48	45	-
Tumor encapsulation									
None	45	66	1.000	73	38	0.692	80	31	0.004
Complete	57	85	-	89	53	-	77	65	-
Tumor differentiation									
I + II	71	105	1.000	113	63	1.000	106	70	0.400
III + IV	81	46	-	49	28	-	51	26	-
TNM stage									
I	65	92	0.693	113	49	0.001	108	49	0.005
III + IV	37	59	-	44	47	-	49	47	-

HCC, hepatocellular carcinoma; HBsAg, hepatitis B surface antigen; HCVAb, hepatitis C virus antibody; AFP, α-fetoprotein; ALT, alanine transaminase; γ-GT, γ-glutamyltransferase; TNM, tumor-node-metastasis. ^#^: The Pearson Chi square test was applied.

**Table 2:** Univariate and multivariate analysis of factors related to OS and TTR of HCC patients in the cohort (*n* = 253).

Variables	OS			TTR				
	Univariate	Multivariate		Univariate		Multivariate		
	*p*	HR	95% CI	*p*	*p*	HR	95% CI	*p*
Age, years (>51 *vs.* ≤51)	0.624	-	-	NA	0.437	-	-	NA
Gender (male *vs.* female)	0.273	-	-	NA	0.990	-	-	NA
HBsAg (positive *vs.* negative)	0.594	-	-	NA	0.389	-	-	NA
HCVAb (positive *vs.* negative)	0.180	-	-	NA	0.307	-	-	NA
Serum AFP, ng/mL (>20 *vs.* ≤20)	<0.001	2.095	1.323–3.318	0.002	0.027	-	-	NS
Serum ALT, U/L (>75 *vs.* ≤75)	0.945	-	-	NA	0.725	-	-	NA
Serum γ-GT, U/L (>54 *vs.* ≤54)	<0.001	1.799	1.124–2.881	0.014	0.001	1.537	1.028–2.298	0.036
Liver cirrhosis (yes *vs.* no)	0.320	-	-	NA	0.751	-	-	NA
Tumor size (cm) (>5 *vs.* ≤5)	<0.001	2.609	1.635–4.162	<0.001	<0.001	1.735	1.151–2.616	0.008
Tumor multiplicity (multiple *vs.* single)	0.362	-	-	NA	0.566	-	-	NA
Tumor encapsulation (none *vs.* complete)	0.493	-	-	NA	0.153	-	-	NA
Tumor differentiation (poor *vs.* well)	<0.001	2.166	1.436–3.267	<0.001	0.008	-	-	NS
Vascular invasion (yes *vs.* no)	<0.001	1.756	1.139–2.709	0.011	<0.001	1.696	1.136–2.531	0.016
TNM stage (III-II *vs.* I ^b^)	0.028	-	-	NS	0.103	-	-	NA
CD68^+^ TAMs (High *vs.* Low ^b^)	0.095	-	-	NA	0.169	-	-	NA
CD86^+^ TAMs (Low *vs.* High ^b^)	0.029	2.178	1.333–3.558	0.002	0.040	1.810	1.181–2.776	0.006
CD206^+^ TAMs (High *vs.* Low ^b^)	0.026	1.584	1.053–2.385	0.027	0.033	1.872	1.065–3.290	0.030
CD86/CD206 signature ^a^	0.002	-	-	0.002	0.004	-	-	0.007
II *vs.* I ^b^	0.020	2.343	1.173–4.681	0.016	0.033	1.872	1.065–3.290	0.029
III *vs.* I ^b^	0.020	2.685	1.218–5.920	0.014	0.047	1.703	0.833–3.483	0.144
IV *vs.* I ^b^	0.002	3.358	1.622–6.952	0.001	0.004	2.377	1.305–4.330	0.005

HCC, hepatocellular carcinoma; OS, overall survival; TTR, time to recurrence; HBsAg, hepatitis B surface antigen; HCVAb, hepatitis C virus antibody; AFP, alpha-fetoprotein; ALT, alanine transaminase; γ-GT, γ-glutamyltransferase; TNM, tumor-node-metastasis; HR, hazard ratio; CI, confidential interval; NA, not applicable; NS, not significant. Univariate analysis was performed by Kaplan–Meier method (log-rank test). Multivariate analysis was calculated using the Cox multivariate proportional hazard regression model with stepwise manner. ^a^ Patients were divided into four groups based on their staining densities of CD86 and CD206 positive TAMs: Group I, high expression of CD86 but low expression of CD206; Group II, both low expressions; Group III, both high expressions; and Group IV, low expression of CD86 but high expression of CD206; ^b^ Control group.

## Supplementary Materials

Supplementary materials can be found at http://www.mdpi.com/1422-0067/17/3/320/s1.
