# Supplementary material for: CD86+/CD206+, Diametrically Polarized Tumor-Associated Macrophages, Predict Hepatocellular Carcinoma Patient Prognosis
Source: Int J Mol Sci. 2016 Mar 1;17(3):320. doi: 10.3390/ijms17030320 (PMC4813183; doi:10.3390/ijms17030320)
Supplement: Supplementary file 1 [file ijms-17-00320-s001.pdf]

Supplementary Materials: CD86$^+$/CD206$^+$, Diametrically Polarized Tumor-Associated Macrophages, Predict Hepatocellular Carcinoma Patient Prognosis

Pingping Dong, Lijie Ma, Longzi Liu, Guangxi Zhao, Si Zhang, Ling Dong, Ruyi Xue and She Chen

Table S1. Descriptive statistics of immunohistochemical variables.

Variable $^\#$	Mean	SD	Median	Range
CD68$^+$ TAMs	64.3	21.6	67.3	8–112
CD86$^+$ TAMs	41.5	19.3	36.7	5–93
CD206$^+$ TAMs	30.5	14.8	33.3	5–78

TAM: Tumor-associated macrophage; SD: Standard deviation. $^\#$: Number of cells per field (×200 magnification).
